# *N*′-(4-Meth­oxy­benzyl­idene)-4-methyl­benzohydrazide

**DOI:** 10.1107/S1600536811049932

**Published:** 2011-11-25

**Authors:** Zeng-Xin Liu

**Affiliations:** a Experimental Center, Linyi University, Linyi 276005, People’s Republic of China

## Abstract

The title compound, C_16_H_16_N_2_O_2_, is the product of the reaction of 4-meth­oxy­benzaldehyde and 4-methyl­benzo­hydrazide. The dihedral angle between the substituted benzene rings is 17.6 (3)° and the meth­oxy C atom is almost coplanar with its attached ring [deviation = 0.019 (4) Å]. In the crystal, mol­ecules are linked by N—H⋯O hydrogen bonds, forming *C*(4) chains propagating along the *b*-axis direction.

## Related literature

For reference bond lengths, see: Allen *et al.* (1987 ▶). For related strctures, see: Horkaew *et al.* (2011 ▶); Fun *et al.* (2011 ▶); Su *et al.* (2011 ▶); Hashemian *et al.* (2011 ▶); Promdet *et al.* (2011 ▶).
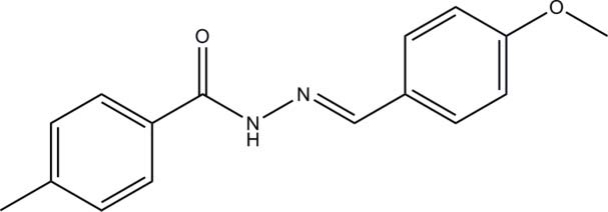


## Experimental

### 

#### Crystal data

C_16_H_16_N_2_O_2_
*M* *_r_* = 268.31
Orthorhombic, 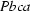

*a* = 12.138 (2) Å
*b* = 8.0580 (16) Å
*c* = 29.320 (3) Å
*V* = 2867.7 (8) Å^3^
*Z* = 8
Mo *K*α radiation
μ = 0.08 mm^−1^
*T* = 298 K
0.17 × 0.13 × 0.12 mm

#### Data collection

Bruker SMART 1K CCD diffractometer
Absorption correction: multi-scan (*SADABS*; Sheldrick, 1996 ▶) *T*_min_ = 0.986, *T*_max_ = 0.990
20247 measured reflections
3098 independent reflections
1427 reflections with *I* > 2σ(*I*)
*R*_int_ = 0.137

#### Refinement

*R*[*F* ^2^ > 2σ(*F* ^2^)] = 0.082
*wR*(*F* ^2^) = 0.212
*S* = 1.01
3098 reflections
186 parameters
1 restraint
H atoms treated by a mixture of independent and constrained refinement
Δρ_max_ = 0.16 e Å^−3^
Δρ_min_ = −0.24 e Å^−3^

### 

Data collection: *SMART* (Bruker, 2007 ▶); cell refinement: *SAINT* (Bruker, 2007 ▶); data reduction: *SAINT*; program(s) used to solve structure: *SHELXS97* (Sheldrick, 2008 ▶); program(s) used to refine structure: *SHELXL97* (Sheldrick, 2008 ▶); molecular graphics: *SHELXTL* (Sheldrick, 2008 ▶); software used to prepare material for publication: *SHELXL97*.

## Supplementary Material

Crystal structure: contains datablock(s) I, global. DOI: 10.1107/S1600536811049932/hb6527sup1.cif

Structure factors: contains datablock(s) I. DOI: 10.1107/S1600536811049932/hb6527Isup2.hkl

Supplementary material file. DOI: 10.1107/S1600536811049932/hb6527Isup3.cml

Additional supplementary materials:  crystallographic information; 3D view; checkCIF report

## Figures and Tables

**Table 1:** Hydrogen-bond geometry (Å, °)

*D*—H⋯*A*	*D*—H	H⋯*A*	*D*⋯*A*	*D*—H⋯*A*
N1—H1⋯O1^i^	0.90 (1)	1.97 (1)	2.870 (4)	176 (3)

Symmetry code: (i) 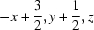.

**Fig. 1.**
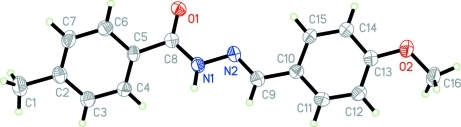
The molecular structure of the title compound, showing 30% probability displacement ellipsoids.

**Fig. 2.**
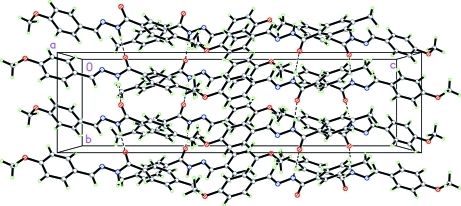
The molecular packing of the title compound, showing the N—H···O hydrogen-bonds (dashed lines).

## supplementary crystallographic information

### Comment

Recently, the compounds derived from the condensation reaction of carbonyl-containing compounds with substituted benzohydrazides have received considerable attention. In this paper, the title new compound, derived from the reaction of 4-methoxybenzaldehyde with 4-methylbenzohydrazide, is reported.

The molecule of the compound, Fig. 1, displays a *trans*-configuration about the C9 ═N2 bond. The torsion angle of C8—N1—N2—C9 is 2.3 (3)°. The dihedral angle between the C2—C7 and C10—C15 benzene rings is 17.6 (3)°, indicating the molecule of the compound is twisted. Overall, the bond distances are within normal values (Allen *et al.*, 1987), and are comparable with those reported in similar compounds (Horkaew *et al.*, 2011; Fun *et al.*, 2011; Su *et al.*, 2011; Hashemian *et al.*, 2011; Promdet *et al.*, 2011). In the crystal, molecules are linked by N—H···O hydrogen bonds (Table 1) to form C(4) chains along the *b* axis (Fig. 2).

### Experimental

The title compound was synthesized by the reaction of 4-methoxybenzaldehyde (1 mmol, 0.14 g) with 4-methylbenzohydrazide (1 mmol, 0.15 g) in absolute methanol (30 ml) at ambient condition. Colorless prism-shaped single crystals were obtained by slow evaporation of the solution at room temperature after several days.

### Refinement

The amide H atom was located in a difference map and was refined isotropically, with N—H = 0.90 (1) Å. The remaining H atoms were positioned geometrically and allowed to ride on their parent atoms, with C—H = 0.93 Å for aromatic and CH and 0.96 Å for CH_3_ atoms. The *U*_iso_ values were constrained to be 1.5*U*_eq_ of the carrier atom for methyl H atoms and 1.2*U*_eq_ for the remaining H atoms. A rotating group model was used for the methyl groups.

### Crystal data

**Table:**

C_16_H_16_N_2_O_2_	*D*_x_ = 1.243 Mg m^−^^3^
*M**_r_* = 268.31	Mo *K*α radiation, λ = 0.71073 Å
Orthorhombic, *P**b**c**a*	Cell parameters from 883 reflections
*a* = 12.138 (2) Å	θ = 2.2–24.3°
*b* = 8.0580 (16) Å	µ = 0.08 mm^−^^1^
*c* = 29.320 (3) Å	*T* = 298 K
*V* = 2867.7 (8) Å^3^	Prism, colorless
*Z* = 8	0.17 × 0.13 × 0.12 mm
*F*(000) = 1136	

### Data collection

**Table:**

Bruker SMART 1K CCD diffractometer	3098 independent reflections
Radiation source: fine-focus sealed tube	1427 reflections with *I* > 2σ(*I*)
graphite	*R*_int_ = 0.137
ω scan	θ_max_ = 27.0°, θ_min_ = 1.4°
Absorption correction: multi-scan (*SADABS*; Sheldrick, 1996)	*h* = −15→15
*T*_min_ = 0.986, *T*_max_ = 0.990	*k* = −10→10
20247 measured reflections	*l* = −36→36

### Refinement

**Table:**

Refinement on *F*^2^	Primary atom site location: structure-invariant direct methods
Least-squares matrix: full	Secondary atom site location: difference Fourier map
*R*[*F*^2^ > 2σ(*F*^2^)] = 0.082	Hydrogen site location: inferred from neighbouring sites
*wR*(*F*^2^) = 0.212	H atoms treated by a mixture of independent and constrained refinement
*S* = 1.01	*w* = 1/[σ^2^(*F*_o_^2^) + (0.0711*P*)^2^] where *P* = (*F*_o_^2^ + 2*F*_c_^2^)/3
3098 reflections	(Δ/σ)_max_ < 0.001
186 parameters	Δρ_max_ = 0.16 e Å^−^^3^
1 restraint	Δρ_min_ = −0.24 e Å^−^^3^

### Special details

**Table:**

Geometry. All e.s.d.'s (except the e.s.d. in the dihedral angle between two l.s. planes) are estimated using the full covariance matrix. The cell e.s.d.'s are taken into account individually in the estimation of e.s.d.'s in distances, angles and torsion angles; correlations between e.s.d.'s in cell parameters are only used when they are defined by crystal symmetry. An approximate (isotropic) treatment of cell e.s.d.'s is used for estimating e.s.d.'s involving l.s. planes.
Refinement. Refinement of *F*^2^ against ALL reflections. The weighted *R*-factor *wR* and goodness of fit *S* are based on *F*^2^, conventional *R*-factors *R* are based on *F*, with *F* set to zero for negative *F*^2^. The threshold expression of *F*^2^ > σ(*F*^2^) is used only for calculating *R*-factors(gt) *etc*. and is not relevant to the choice of reflections for refinement. *R*-factors based on *F*^2^ are statistically about twice as large as those based on *F*, and *R*- factors based on ALL data will be even larger.

### Fractional atomic coordinates and isotropic or equivalent isotropic displacement parameters (Å^2^)

**Table:**

	*x*	*y*	*z*	*U*_iso_*/*U*_eq_	
N1	0.7556 (2)	0.2199 (4)	0.15333 (9)	0.0515 (7)	
N2	0.7692 (2)	0.1612 (3)	0.10929 (8)	0.0503 (7)	
O1	0.62721 (19)	0.0243 (3)	0.16866 (7)	0.0543 (7)	
O2	0.9212 (2)	0.0558 (3)	−0.09555 (8)	0.0715 (8)	
C1	0.6336 (3)	0.3773 (6)	0.36707 (12)	0.0858 (13)	
H1A	0.5711	0.4498	0.3692	0.129*	
H1B	0.6987	0.4356	0.3766	0.129*	
H1C	0.6224	0.2827	0.3865	0.129*	
C2	0.6473 (3)	0.3199 (4)	0.31818 (11)	0.0562 (9)	
C3	0.7427 (3)	0.3503 (5)	0.29457 (11)	0.0623 (10)	
H3	0.7994	0.4082	0.3088	0.075*	
C4	0.7566 (3)	0.2965 (4)	0.25005 (11)	0.0569 (10)	
H4	0.8226	0.3169	0.2350	0.068*	
C5	0.6726 (3)	0.2127 (4)	0.22787 (10)	0.0465 (8)	
C6	0.5756 (3)	0.1825 (5)	0.25146 (11)	0.0571 (10)	
H6	0.5185	0.1248	0.2375	0.069*	
C7	0.5638 (3)	0.2383 (5)	0.29572 (12)	0.0634 (10)	
H7	0.4975	0.2203	0.3108	0.076*	
C8	0.6825 (3)	0.1443 (4)	0.18108 (11)	0.0458 (8)	
C9	0.8368 (3)	0.2430 (4)	0.08500 (11)	0.0519 (9)	
H9	0.8723	0.3346	0.0975	0.062*	
C10	0.8597 (3)	0.1963 (4)	0.03814 (11)	0.0465 (8)	
C11	0.9435 (3)	0.2702 (4)	0.01397 (11)	0.0574 (10)	
H11	0.9854	0.3518	0.0282	0.069*	
C12	0.9679 (3)	0.2282 (4)	−0.03048 (11)	0.0596 (10)	
H12	1.0257	0.2795	−0.0458	0.072*	
C13	0.9054 (3)	0.1093 (4)	−0.05162 (11)	0.0539 (9)	
C14	0.8201 (3)	0.0326 (5)	−0.02874 (11)	0.0638 (10)	
H14	0.7784	−0.0489	−0.0431	0.077*	
C15	0.7972 (3)	0.0773 (4)	0.01531 (11)	0.0578 (10)	
H15	0.7386	0.0270	0.0304	0.069*	
C16	1.0064 (3)	0.1320 (5)	−0.12119 (12)	0.0757 (12)	
H16A	0.9913	0.2484	−0.1243	0.114*	
H16B	1.0102	0.0819	−0.1509	0.114*	
H16C	1.0755	0.1172	−0.1057	0.114*	
H1	0.790 (3)	0.316 (3)	0.1592 (11)	0.080*	

### Atomic displacement parameters (Å^2^)

**Table:**

	*U*^11^	*U*^22^	*U*^33^	*U*^12^	*U*^13^	*U*^23^
N1	0.0616 (19)	0.0518 (19)	0.0410 (15)	−0.0018 (15)	0.0025 (14)	−0.0037 (14)
N2	0.0576 (17)	0.0523 (18)	0.0410 (16)	0.0043 (14)	0.0019 (14)	−0.0043 (14)
O1	0.0599 (14)	0.0491 (15)	0.0539 (15)	−0.0044 (12)	0.0005 (11)	−0.0067 (12)
O2	0.0843 (19)	0.080 (2)	0.0499 (15)	−0.0157 (15)	0.0173 (13)	−0.0074 (13)
C1	0.091 (3)	0.106 (4)	0.060 (3)	0.003 (3)	0.008 (2)	−0.019 (2)
C2	0.065 (2)	0.057 (2)	0.047 (2)	0.0089 (19)	−0.0014 (18)	0.0015 (17)
C3	0.067 (3)	0.074 (3)	0.046 (2)	−0.008 (2)	0.0000 (19)	0.0008 (19)
C4	0.055 (2)	0.066 (3)	0.049 (2)	−0.0078 (18)	0.0022 (17)	−0.0012 (19)
C5	0.051 (2)	0.046 (2)	0.0420 (19)	0.0092 (16)	0.0036 (16)	0.0000 (16)
C6	0.047 (2)	0.066 (3)	0.058 (2)	0.0035 (18)	−0.0007 (17)	−0.0057 (19)
C7	0.053 (2)	0.077 (3)	0.061 (2)	0.010 (2)	0.0118 (18)	−0.005 (2)
C8	0.048 (2)	0.042 (2)	0.048 (2)	0.0080 (18)	−0.0020 (16)	0.0031 (16)
C9	0.056 (2)	0.051 (2)	0.049 (2)	0.0036 (18)	−0.0009 (16)	−0.0015 (17)
C10	0.0493 (19)	0.040 (2)	0.050 (2)	0.0008 (16)	0.0016 (16)	0.0028 (16)
C11	0.061 (2)	0.052 (2)	0.059 (2)	−0.0082 (18)	0.0001 (19)	−0.0059 (18)
C12	0.060 (2)	0.060 (3)	0.059 (2)	−0.0115 (19)	0.0126 (18)	−0.0009 (19)
C13	0.056 (2)	0.057 (2)	0.049 (2)	0.0008 (18)	0.0017 (18)	−0.0012 (18)
C14	0.065 (2)	0.073 (3)	0.053 (2)	−0.018 (2)	0.0060 (18)	−0.0021 (19)
C15	0.062 (2)	0.062 (3)	0.050 (2)	−0.0145 (19)	0.0089 (18)	0.0023 (18)
C16	0.091 (3)	0.084 (3)	0.053 (2)	−0.002 (2)	0.024 (2)	0.003 (2)

### Geometric parameters (Å, °)

**Table:**

N1—C8	1.349 (4)	C6—C7	1.381 (4)
N1—N2	1.385 (3)	C6—H6	0.9300
N1—H1	0.899 (10)	C7—H7	0.9300
N2—C9	1.271 (4)	C9—C10	1.451 (4)
O1—C8	1.233 (4)	C9—H9	0.9300
O2—C13	1.372 (4)	C10—C11	1.375 (4)
O2—C16	1.418 (4)	C10—C15	1.394 (4)
C1—C2	1.515 (5)	C11—C12	1.379 (4)
C1—H1A	0.9600	C11—H11	0.9300
C1—H1B	0.9600	C12—C13	1.370 (4)
C1—H1C	0.9600	C12—H12	0.9300
C2—C3	1.371 (5)	C13—C14	1.380 (4)
C2—C7	1.376 (5)	C14—C15	1.369 (4)
C3—C4	1.385 (4)	C14—H14	0.9300
C3—H3	0.9300	C15—H15	0.9300
C4—C5	1.385 (4)	C16—H16A	0.9600
C4—H4	0.9300	C16—H16B	0.9600
C5—C6	1.388 (4)	C16—H16C	0.9600
C5—C8	1.483 (4)		
			
C8—N1—N2	119.1 (3)	O1—C8—C5	121.4 (3)
C8—N1—H1	126 (2)	N1—C8—C5	116.3 (3)
N2—N1—H1	115 (2)	N2—C9—C10	121.3 (3)
C9—N2—N1	114.9 (3)	N2—C9—H9	119.3
C13—O2—C16	117.6 (3)	C10—C9—H9	119.3
C2—C1—H1A	109.5	C11—C10—C15	116.9 (3)
C2—C1—H1B	109.5	C11—C10—C9	121.2 (3)
H1A—C1—H1B	109.5	C15—C10—C9	121.9 (3)
C2—C1—H1C	109.5	C10—C11—C12	122.7 (3)
H1A—C1—H1C	109.5	C10—C11—H11	118.7
H1B—C1—H1C	109.5	C12—C11—H11	118.7
C3—C2—C7	117.8 (3)	C13—C12—C11	118.7 (3)
C3—C2—C1	121.0 (3)	C13—C12—H12	120.7
C7—C2—C1	121.2 (3)	C11—C12—H12	120.7
C2—C3—C4	121.5 (3)	C12—C13—O2	124.5 (3)
C2—C3—H3	119.3	C12—C13—C14	120.6 (3)
C4—C3—H3	119.3	O2—C13—C14	114.9 (3)
C5—C4—C3	120.4 (3)	C15—C14—C13	119.5 (3)
C5—C4—H4	119.8	C15—C14—H14	120.2
C3—C4—H4	119.8	C13—C14—H14	120.2
C4—C5—C6	118.4 (3)	C14—C15—C10	121.6 (3)
C4—C5—C8	123.8 (3)	C14—C15—H15	119.2
C6—C5—C8	117.7 (3)	C10—C15—H15	119.2
C7—C6—C5	119.9 (3)	O2—C16—H16A	109.5
C7—C6—H6	120.0	O2—C16—H16B	109.5
C5—C6—H6	120.0	H16A—C16—H16B	109.5
C2—C7—C6	122.0 (3)	O2—C16—H16C	109.5
C2—C7—H7	119.0	H16A—C16—H16C	109.5
C6—C7—H7	119.0	H16B—C16—H16C	109.5
O1—C8—N1	122.3 (3)		

### Hydrogen-bond geometry (Å, °)

**Table:**

*D*—H···*A*	*D*—H	H···*A*	*D*···*A*	*D*—H···*A*
N1—H1···O1^i^	0.90 (1)	1.97 (1)	2.870 (4)	176 (3)

Symmetry codes: (i) −*x*+3/2, *y*+1/2, *z*.
